# Brown Bear Consumption of Pacific Salmon Varies Greatly Among Individuals but Increases With the Bear's Age and Salmon Availability

**DOI:** 10.1002/ece3.73062

**Published:** 2026-02-12

**Authors:** Thomas P. Quinn, Jennifer H. Stern, N. Grace Henry, Annika K. McFeely, HyeJoo Ro, Blaise Stricker, Liz Voytas, Lisette P. Waits, Jennifer R. Adams, Aaron J. Wirsing

**Affiliations:** ^1^ School of Aquatic and Fishery Sciences University of Washington Seattle Washington USA; ^2^ School of Environmental and Forest Sciences University of Washington Seattle USA; ^3^ Department of Fish and Wildlife Sciences University of Idaho Moscow Idaho USA

**Keywords:** diet, marine‐derived nutrients, omnivory, *Oncorhynchus*, stable isotopes, *Ursus arctos*

## Abstract

As animals age and grow, their diet often changes as food demand increases, and because their size and social dominance may allow them to capture and consume larger and different prey. Diet also can vary between sexes and individuals. In this study we used stable isotope analysis of hair samples collected from free‐living brown bears, *Ursus arctos* , identified by DNA analysis, in an area with easy access to Pacific salmon (*Oncorhynchus* sp.). The goals were to test the hypotheses that salmon consumption would increase as bears aged, be higher in males than females, and higher in bears with access to more than fewer salmon, in the context of the level of variation among individuals. Samples (*n* = 165 from 85 different bears) were collected from six streams, three on each side of Lake Aleknagik, Alaska from 2012 to 2022 (missing only 2020) and processed for isotopes of N and C. These data confirmed our predictions of greater salmon consumption as individuals aged and in those sampled along streams with higher salmon densities. However, inferred salmon consumption varied greatly among bears and did not differ consistently between sexes, though far more females were detected than males. Thus, at the population level, salmon availability affected inferred consumption by bears, and average consumption increased as the bear aged and presumably grew larger with higher social status. Nevertheless, some individuals consumed little salmon, though the hair samples were collected from bears traveling along small streams with dense salmon populations where predation is common. They may have been using the streams primarily as traveling corridors rather than foraging areas, despite the abundance of salmon. In any case, the varied reliance on salmon, without sex bias, observed here differs from systems where males consume more salmon and some females avoid salmon‐bearing streams.

## Graphical abstract

Hair was sampled in a noninvasive manner from free‐living brown bears in Alaska and processed for genetic identification and stable isotopes. These data revealed great individual variation in bear reliance on salmon, despite being sampled along streams full of salmon. Bear reliance on salmon increased as they aged, but did not differ consistently between males and females.

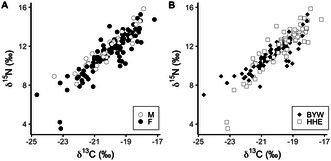

## 1 Introduction

Many animals consume varied diets, optimizing the balance between nutritional needs, food availability, and other considerations such as competition, predator avoidance, and the capacity to obtain and consume the food items. As individuals grow, their capacity to capture different and often larger prey can increase, and their vulnerability to competition and predation may decrease, and habitat use may shift (Werner and Gilliam 1984). For example, salmon and trout diets change as they grow, as their increased gape allows them to consume larger prey (Grey 2001; Keeley and Grant 2001); similar patterns are observed in many fishes (Stallings et al. 2023), amphibians (Zerba and Collins 1992), and birds (Edwards Jr. 1989). In addition to the trophic shift often observed with ontogeny, there is also considerable variation among individuals in diet, even among those with access to the same prey (Edwards Jr. 1989; Kim et al. 2012; Stallings et al. 2023). Some diet variation is related to differences in foraging between males and females (Tennessen et al. 2023), but social position and other behavioral factors are also involved (Harrison et al. 2017; Johnsson and Näslund 2018; Hertel et al. 2020). Indeed, individual variation, including foraging patterns and diet, is a fundamental aspect of behavioral ecology (Bolnick et al. 2003; Mittelbach et al. 2014).

Notwithstanding the importance of diet for the ecology of the consumer and its forage or prey, practical or ethical considerations may preclude studies based on observed predation events, fecal samples, or stomach content analysis of dead or euthanized individuals. Consequently, stable isotope ratios of carbon (^13^C / ^12^C) and nitrogen (^15^N / ^14^N) are commonly used to infer dietary reliance on certain foods and overall trophic position. Nitrogen isotopic values (δ^15^N) are associated with trophic position (i.e., higher in predators than in their prey) and carbon isotope values (δ^13^C) depend on carbon sources (Minagawa and Wada 1984; Vander Zanden and Rasmussen 1999; Post 2002). Such studies have revealed variation in foraging between sexes and individuals in many animal populations (Urton and Hobson 2005; Samarra et al. 2017; Scholz et al. 2020; LaRoche et al. 2021; Dias et al. 2023). Outside of captive settings, however, isotopic studies rarely offer the opportunity to track the diets of individuals as they age, particularly in large vertebrates, owing to the challenges of reliable resampling. To address this knowledge gap, we used stable isotope analysis of hair samples spanning as many as 10 years from individual, free‐living brown bears, *Ursus arctos* , to explore among‐ and within‐individual patterns of reliance on Pacific salmon, *Oncorhynchus* sp., in southwest Alaska, USA.

Brown bears and black bears, *U. americanus*, consume a wide variety and size range of aquatic and terrestrial vertebrates, invertebrates, and plants (Jacoby et al. 1999; Mowat and Heard 2006; Gunther et al. 2014; McLaren et al. 2021). They eat small prey including army cutworm moths ( *Euxoa auxiliaris* ) (White et al. 1998) and ants (Swenson et al. 1999), as well as ungulates, and in intertidal areas they may eat amphipods and Pacific herring ( *Clupea pallasii* ) eggs (Fox et al. 2015), clams (Smith and Partridge 2004), and scavenge dead whales (Lewis and Lafferty 2014). Despite their dietary breadth, brown bears are strongly inclined to consume Pacific salmon when salmon are available (Mowat and Heard 2006; Adams et al. 2017; Mangipane et al. 2020). Pacific salmon is a valuable resource for brown bears because their predictable seasonal availability and high lipid content allow bears to store energy needed for denning, parturition, and nursing (Hilderbrand, Jenkins, et al. 1999). The consumption of protein and lipid‐dense meats can increase fitness by increasing litter size as well as greater muscle and fat development (Hilderbrand, Schwartz, et al. 1999). Robbins et al. (2004) concluded, “Although brown bear populations exist without salmon, individuals in these populations are smaller, reproduce less often, and exist at densities 1/50th of those occurring in salmon‐feeding populations.” [p. 166].

Despite the many fitness benefits of a high protein and fat, not all bears necessarily consume salmon and terrestrial meat to the same extent. Large bears rely more heavily than smaller bears on meat, but the latter can grow efficiently on a diverse diet including more plant material (Robbins et al. 2007). Furthermore, variable seasonal salmon availability may limit opportunities for large bears to increase meat consumption until salmon are present (Van Daele et al. 2013). Moreover, the risk of infanticide can cause mothers with cubs to avoid areas where large males dominate access to salmon (Ben‐David et al. 2004). As a result of such caution and their smaller size (Hilderbrand, Schwartz, et al. 1999; Bartareau et al. 2011), females often have lower inferred consumption of salmon (Van Daele et al. 2013; Matsubayashi et al. 2014; Adams et al. 2017; Finnegan et al. 2023) and generally lower trophic position than co‐occurring male bears (Hobson et al. 2000; Careddu et al. 2021; Pauly et al. 2026). However, males do not always consume more terrestrial meat and salmon than females (Fortin et al. 2007), especially if salmon are readily available (Mowat and Heard 2006), and inferred diets can vary greatly among individuals (Mangipane et al. 2018; Ro et al. 2021). Because the extent of dietary differences among individuals and between males and females may depend on local prey density, inferred salmon consumption from stable isotope data necessitates detailed information on salmon availability in the specific areas where the bears foraged and repeated sampling of individuals.

The overall goal of this multi‐year study was to test a series of predictions regarding brown bear consumption of Pacific salmon. To do so, we took advantage of the distinctive stable isotope signature of salmon from their reliance on marine foodwebs (Johnson and Schindler 2009) compared to the chief alternative food sources: terrestrial meat and plants (Hilderbrand et al. 1996; Belant et al. 2006; Jimbo et al. 2022). First, we predicted that salmon consumption would be higher where salmon abundance and predation were higher (i.e., more salmon killed each year) than in a nearby area where salmon abundance and predation were lower. The null hypothesis, that salmon consumption was similar between areas, might occur if salmon were sufficiently abundant to meet the bears' needs. Second, we predicted that males would show higher and less variable salmon consumption than females, as the latter might include more diverse foraging patterns owing to their smaller size and maternal care. Our third prediction was that salmon consumption would increase as individuals aged because associated increases in size, experience, and/or social position should improve access to salmon.

## 2 Methods

### 2.1 Study Site

**FIGURE 1:**
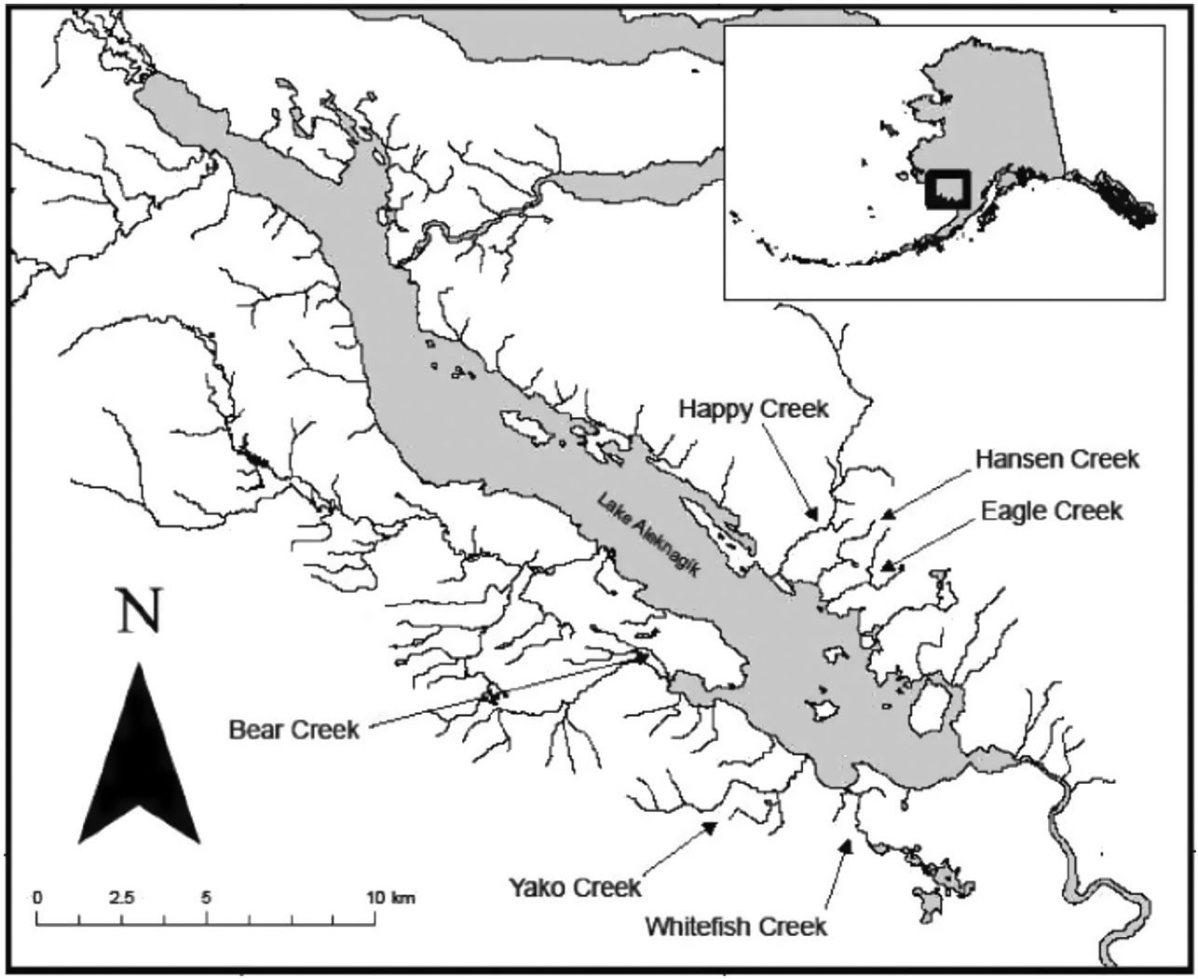
Map of Lake Aleknagik in Alaska, USA showing the six streams where wires were deployed to sample hair from brown bears for DNA and stable isotope analysis (from Ro et al. 2020).

Sampling for this study was conducted on six streams flowing into Lake Aleknagik, in the Wood River system in southwestern Alaska (Figure 1). These and other streams in the basin have been studied for decades as part of a larger sockeye salmon ( *O. nerka* ) ecology and management program (Hilborn et al. 2003), and bear predation has been studied for over 30 years (Quinn et al. 2001, 2003, 2017). These and other streams in this system are used annually for spawning by sockeye salmon (Quinn et al. 2017) and much lower numbers of other salmon species (Pess et al. 2014). The six streams sampled in the present study are small (< 5 m wide and 30 cm deep) and lack the woody debris and cover that might provide shelter from predation; consequently, typically 20%–50% of the sockeye salmon are killed by bears before death from senescence (Quinn et al. 2017).

Happy, Hansen, and Eagle creeks are on the northeast side of the lake and Yako, Whitefish, and Bear creeks are on the southwest side, with no more than a few km of shoreline separating the trios of streams on each side of the lake (Figure 1). We refer to these as two foraging neighborhoods, as prior research (Wirsing et al. 2018) revealed that brown bears commonly move among streams within neighborhoods but very seldom cross the lake to the other neighborhood. Spawning follows a regular pattern from mid‐July to late August: earliest in Happy Creek, followed by Hansen, Yako, and Bear creeks with similar timing, and spawning is latest in Eagle and Whitefish creeks. Spawning timing overlaps considerably between streams and sides of the lake, especially in the first three weeks of August (Lin et al. 2008). The streams are in roadless areas beyond the village of Aleknagik (59.275° N, 158.625° W, population 109 based on the most recent US census data: http://censusreporter.org/profiles/16000US0201420‐aleknagik‐ak/). No logging, farming, or other land use activities occur near the streams, and there are no recreational fishing or other activities during the summer besides our surveys.

### 2.2 Bear Hair Collection and Sample Selection

Bear hair was collected annually from 2012 to 2019, and 2021 to 2022 (the COVID‐19 pandemic precluded sampling in 2020). We stretched barbed wire across each stream at two discrete locations that averaged 700 m apart and anchored it to trees on either side of the stream at the recommended height of ca. 0.5 m above the streambed (Quinn et al. 2022). The wires were checked every two days, and any hair samples were collected and the data (stream, wire, barb number, date, and time) recorded on and stored in an envelope until it could be processed.

The deployment of wires was designed to match the timing of spawning in these streams (Quinn et al. 2014), and the samples retained for analysis were broadly distributed from 16 July to 28 August. More hair samples were collected than could be processed for genetic analysis, so we prioritized specimens based on apparent quality and especially the presence of one or more hair follicles to optimize the likelihood of successful individual identification (Wirsing et al. 2020). Hair samples were also chosen to broaden our spatial and temporal coverage. Samples from different dates, streams, and wires were prioritized over adjacent specimens from the same wire, likely left simultaneously by the same bear. To address these criteria, samples varied in the number and length of hairs and presence of follicles (Wirsing et al. 2020). Genetic analysis, conducted at the University of Idaho, confirmed that all samples were from brown, not black bears, and assigned each sample to a specific bear and its sex. DNA was extracted in a facility dedicated to low‐quality DNA samples using the DNeasy Blood and Tissue Kit (Qiagen Inc.). A genotype was generated for each sample in a multiplex polymerase chain reaction (PCR) using 10 nuclear DNA microsatellite loci and a sex locus. Each sample was amplified two to four times to minimize and detect genotyping errors. Consensus genotypes were obtained following the rule that each allele needed to be observed twice at each locus, and a sample had to contain a consensus genotype at eight or more loci to be included in the matching analysis using the software Genelex (Peakall and Smouse 2006).

Once each specimen was identified, we conducted stable isotope analysis on a subsample, following standard procedures described by Ro et al. (2021). Samples were rinsed in 2:1 chloroform: methanol to remove excess debris and lipid, and then dried at 50°C. Hair samples were then homogenized by cutting them into < 1 cm segments. All samples were weighed into tin capsules (3 × 5 mm) and run for δ^13^C and δ^15^N compositions. Our initial use of the hair samples for stable isotope analysis was based on 77 samples from 31 bears from the years 2012–2015, to assess the overall reliance on Pacific salmon by the bears, variation among individuals, and between males and females (Ro et al. 2021). The main finding was the variation among individuals; effects of bear sex or salmon abundance on inferred consumption were not detected. Those specimens were combined with those collected subsequently for the current analysis because all were similarly collected, processed, and analyzed. Our selection of the current set of samples was designed to address our three hypotheses. Thus, we tried to (1) extend the number of years when individuals were sampled to allow us to assess the effects of minimum age, (2) balance the samples from males and females, (3) balance the samples from the two sides of the lake in multiple years, and (4) include samples from the same bear over multiple dates within a season. It was not possible to optimize all these objectives, but we expanded the data reported by Ro et al. (2021) from 4 to 10 years of sampling, 77 to 165 samples, 31 to 70 different bears, and a span of up to 10 years between the first and most recent samples from an individual bear.

These samples were analyzed at the University of California Davis Stable Isotope Facility (Davis, California) using a PDZ Europa ANCA‐GSL elemental analyzer and PDZ Europa 20–20 isotope ratio mass spectrometer. Isotopic compositions are expressed in standard delta notation (‰), which is calculated by the equation δX = [(*R*_sample_/*R*_standard_) × 1] × 1000; where δX = ^13^C or ^15^N, *R* = ratio of ^13^C/^12^C or ^15^N/^14^N, and standards are Vienna Pee Dee Belemnite (VPDB) and air, respectively. The mean standard deviation for internal standards ranged from 0.03‰ to 0.09‰ for δ^13^C and 0.03‰–0.19‰ for δ^15^N. Sample precision measured by the variance between duplicates was 0.41‰ for δ^13^C and 0.47‰ for δ^15^N. Some scientists (e.g., Finnegan et al. 2023) adjusted the δ^13^C values in studies over multiple years to account for the global decrease in δ^13^C of atmospheric CO_2_ from the burning of fossil fuels. We report the results of our analyses conducted on the observed, unadjusted values (provided in Table S1), to facilitate comparisons to other studies that did not adjust their data, but the conclusions remained the same when statistical tests and models were run with adjusted values.

### 2.3 Estimation of Salmon Abundance and Consumption

Sockeye and other salmon species have been counted using standard methods for many decades in many streams in the Wood River system, including the six streams where we obtained hair samples from brown bears, as described previously (Quinn et al. 2001, 2017). Briefly, around the peak date of spawning, staff walk the stream counting all live and dead salmon, and categorize the dead salmon by mode of death (primarily bear predation or senescence). As some salmon enter the stream after the counts are completed and others are removed by bears from the stream before they are counted (Quinn et al. 2009), survey counts underestimate the total number of salmon in each stream. Nevertheless, they capture the overall patterns of abundance among streams and years. The survey data were also used to estimate the proportion of salmon killed each year in each stream, using a method detailed in Quinn et al. (2001). These stream‐specific estimates were combined to estimate the number of salmon killed for each neighborhood and year. Many carcasses are not entirely consumed and so the number killed is not the same as the biomass of salmon eaten (Lincoln and Quinn 2019), but these data provide a sufficiently accurate index for the present purposes. The salmon estimates are presented for the years when hair samples were obtained (2012–2022) and 2011, because hair sampled in 2012 might reflect in part salmon eaten in 2011 (Table 1).

**TABLE 1:** Annual estimates of the number of adult sockeye salmon available as prey for brown bears and the estimated total number they killed in the two foraging neighborhoods of Lake Aleknagik where hair samples were obtained: Happy, Hansen, and Eagle creeks on the northeast side of the lake, and Bear, Whitefish, and Yako creeks on the southwest side.

Year	Northeast		Southwest	
	No. of salmon	No. killed	No. of salmon	No. killed
2011	14,018	9524	3858	2220
2012	10,764	7023	1787	1010
2013	7144	3976	4704	1915
2014	89,483	4650	23,695	3491
2015	20,833	9639	5681	2964
2016	18,830	7391	4461	1845
2017	46,628	5682	26,407	4221
2018	30,101	3782	21,904	3670
2019	18,064	9158	10,369	3805
2020	26,086	8270	7855	3006
2021	29,823	4903	15,515	4229
2022	31,001	3367	28,265	2150
Average	28,564.6	6447.1	12,875.0	2877.3

### 2.4 Statistical Methods

Of necessity, the 165 samples were not balanced with respect to all variables of interest. The 70 different bears included 27 males, 42 females, and 1 of unknown sex. After excluding the bear of unknown sex, we calculated means and standard deviations using all the samples, separated by sex and neighborhood but pooling among years and individuals, to provide an overall summary of the data (Table 2). We compared mean isotope values between groups using Welch's two‐sample *t*‐tests and evaluated statistical significance at *α* = 0.05. To assess population‐level patterns while retaining all sampled individuals, we fit linear mixed‐effects models to the full dataset. We modeled δ^13^C or δ^15^N with fixed effects of sex and foraging neighborhood, used as a spatial proxy for salmon availability, given uncertainty in the timing of hair growth. We also included the date when the sample was obtained with the understanding that those collected in mid‐summer might include hairs produced in the present and the previous year, and the proportions might affect isotopic signatures (Jimbo et al. 2020; Stern et al. 2025). We included crossed random intercepts for bear identity and year to account for repeated sampling of individuals across multiple years and interannual variation.

**TABLE 2:** Average (SD in parentheses) δ^15^N and δ^13^C values for brown bear hair collected from two foraging neighborhoods of Lake Aleknagik between 2012 and 2022.

Sex	δ^15^N mean (SD)		δ^13^C mean (SD)	
	Southwest	Northeast	Southwest	Northeast
Females	10.9 (1.5)	12.0 (2.4)	−20.4 (1.3)	−19.6 (1.3)
Males	11.4 (1.7)	11.8 (1.6)	−20.3 (1.4)	−20.0 (1.1)
Total	11.0 (1.6)	11.9 (2.1)	−20.4 (1.3)	−19.7 (1.3)

*Note:* Sample sizes, representing separate samples over multiple dates, years, and individual bears, were as follows: 67 from females on the southwest side and 53 from females on the northeast side, 17 from males on the southwest side and 27 from males on the northeast side, for a total of 164 samples.

Because our primary focus was on individual variation and the consistency or changes among years in isotopic signatures, we then reduced the data set to include only individuals sampled in more than 1 year. This resulted in 117 samples from 34 different bears, including 100 samples from 29 females and 17 samples from 5 males. We did not know the bear's age when the samples were obtained, and the earliest sample processed for stable isotopes was not always the first one collected from that bear. We expressed the year when each sample was obtained for stable isotope analysis as the minimum age, relative to the first sample obtained. For example, hair obtained in 2014 from a bear first identified by DNA in 2012 would have a minimum age of 3, indicating that it was in at least its third year of life when that sample was collected. We calculated two predictors from minimum age to distinguish between within‐individual changes associated with aging and differences among bears sampled at different ages, following the general statistical approach of van de Pol and Wright (2009). For each bear, we calculated the average minimum age across all sampling years and subtracted this value from the observed minimum age at each sampling event. This yielded a within‐individual minimum age change term, referred to as a relative age, allowing us to test whether individual bears' isotope signatures changed as they aged over the study period.

For the subset of bears sampled in multiple years, we fit linear mixed models for δ^13^C and δ^15^N values to examine within‐individual changes over time. Fixed effects included foraging neighborhood, sex, day‐of‐year, relative age, average minimum age, and the interaction between sex and relative age. All models included bear identification and sampling year as crossed random effects to account for individual variation and the uncertainty of which year a hair sample represents. We then used the Akaike Information Criterion, corrected for small sample size (AICc), to compare candidate models against the global model (Akaike 1974). Candidate models included versions with and without the interaction between sex and relative age, without average minimum age or sex, and without any age‐related covariates. This approach allowed us to assess the relative support for model terms, while interpreting results primarily from the global model. We calculated the marginal and conditional deviance explained (*R* ^2^) for the global models using the ‘r2glmm’ package (Jaeger 2017). We calculated the relative amount of variance explained by each predictor (partial *R* ^2^) using the Nakagawa and Schielzeth (2013) approach.

All statistical tests and models were run using R v.4.4.2 (R Core Team 2024). Models were fit using the lmer() function (REML = FALSE) in the lme4 package (Bates et al. 2015). We estimated 95% confidence intervals for all model parameters using the confint() function in R. Intervals for fixed effects were calculated using the profile likelihood method, which accounts for the sampling distribution of each parameter. Visual inspection of residual plots and normal Q‐Q plots confirmed that residuals met assumptions of normality and homoscedasticity for all models.

## 3 Results

Testing the hypothesized effect of salmon abundance on consumption by brown bears hinged on differences in salmon abundance between regions, and this was the case. During the study period (2011–2022), the northeast trio of streams had more spawning sockeye salmon, and more were killed by bears compared to the southwest trio of streams in every year (mean abundance: 28,564.6 vs. 12,875.0; mean number killed: 6447.1 vs. 2877.3; Table 1). The predation rate (i.e., percentage killed out of all salmon counted) was similar between the streams, averaging 34.5% in the northeast area and 33.8% in the southeast. Predation rate varied among years, showing the decline with salmon abundance reported earlier (Quinn et al. 2003, 2017).

**FIGURE 2:**
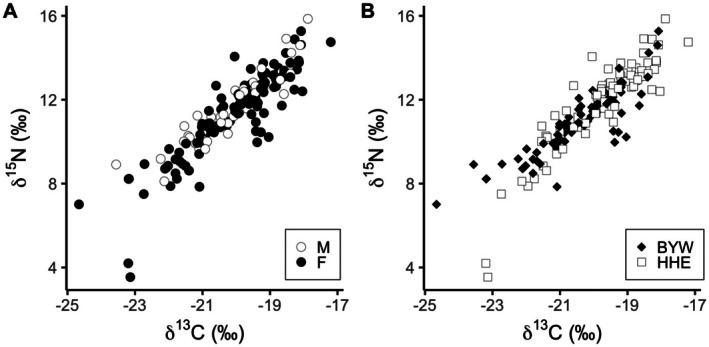
Biplots of carbon and nitrogen stable isotope values of hair samples from individual brown bears *Ursus arctos* sampled in western Alaska, USA. Panel A shows males (M) as white circles, females (F) in black circles. Panel B shows samples collected from the northeast neighborhood (Happy, Hansen, and Eagle creeks, HHE) as white squares and the southwest neighborhood (Bear, Yako, and Whitefish creeks, BYW) as black diamonds.

Our initial analysis included all hair samples to characterize the overall patterns (Table 2; Figure 2). These data indicated that the samples from the northeast neighborhood (where salmon were more abundant) had higher values of δ^15^N and δ^13^C than those from the southwest neighborhood (*p* < 0.01), and that males and females did not differ (*p* > 0.05). When models were fit to the full dataset to characterize population‐level patterns while retaining all sampled individuals, both δ^15^N and δ^13^C exhibited strong among‐individual variation. Of the random effects, individual accounted for substantially more variance than year in both models (δ^15^N: individual variance = 2.27, year variance = 0.33; δ^13^C: individual variance = 0.78, year variance = 0.21). Isotope values declined seasonally with day‐of‐year (δ^15^N: β = −0.03, 95% CI [−0.05, −0.01]; δ^13^C: β = −0.02, 95% CI [−0.04, −0.01]). Confidence intervals for sex overlapped zero in both models (δ^15^N: β = 0.44, 95% CI [−0.44, 1.34]; δ^13^C: β = −0.0002, 95% CI [−0.57, 0.57]), indicating it was not a significant predictor.

We then proceeded to the analysis of the smaller set of 117 samples, including only those from the 5 males and 29 females sampled in more than 1 year. The highest minimum age observed was 11, and 11 bears were sampled at least 7 years after their first detection.

Across both isotopes, support was concentrated among models that included relative age, foraging neighborhood, and day‐of‐year. Adding sex or the sex‐relative age interaction yielded comparable model support, whereas excluding age‐related covariates resulted in substantially poorer fit (Table 3). The confidence intervals for neighborhood, relative age, and day‐of‐year did not overlap zero in the global models for both δ^13^C and δ^15^N, indicating higher values for samples obtained later in an individual's life and in the northeast foraging neighborhood, and lower values for samples obtained later in the season (Table 4). Foraging neighborhood explained the most variance among the fixed effects in the δ^13^C and δ^15^N models (partial *R* ^2^ values in Table 4). The marginal deviance explained (*R* ^2^_marginal_) for the δ^15^N and δ^13^C models was 0.34 and 0.33, respectively, and the conditional deviance explained (*R* ^2^_conditional_) for the δ^15^N and δ^13^C models was 0.58 and 0.52, respectively.

**TABLE 3:** Model selection to assess variables affecting δ^13^C and δ^15^N values of hair samples from brown bears *Ursus arctos* .

Response	Model	df	AIC_c_	ΔAIC_c_	weight
δ^13^C	*neighborhood* + *relative age* + *avg minimum age* + *day‐of‐year*	8	352.7	0.00	0.48
	*neighborhood* + *relative age* + *day‐of‐year* + *sex*	8	353.5	0.77	0.32
	*neighborhood* + *relative age* + *avg minimum age* + *day‐of‐year* + *sex*	9	355.1	2.35	0.15
	*neighborhood* + *relative age* + *avg minimum age* + *day‐of‐year* + *sex* + (relative age*sex)	10	357.0	4.28	0.06
	*neighborhood* + *day‐of‐year* + *sex*	7	366.0	13.28	0.00
δ^15^N	*neighborhood* + *relative age* + *avg minimum age* + *day‐of‐year*	8	425.5	0.00	0.41
	*neighborhood* + *relative age* + *day‐of‐year* + *sex*	8	425.9	0.31	0.35
	*neighborhood* + *relative age* + *avg minimum age* + *day‐of‐year* + *sex*	9	427.8	2.30	0.13
	*neighborhood* + *relative age* + *avg minimum age* + *day‐of‐year* + *sex* + (relative age*sex)	10	428.3	2.79	0.10
	*neighborhood* + *day‐of‐year* + *sex*	7	436.2	10.61	0.00

*Note:* Models were built using fixed effects of the sex of the sampled bear (sex), within‐individual age change (*relative age*), the average minimum age (*avg minimum age*), the timing of sample collection (day‐of‐year), and foraging neighborhoods of Lake Aleknagik in which the sample was collected (neighborhood). All models included two crossed random intercepts for individual bear and collection year as predictors.

**TABLE 4:** Estimated regression parameters (β), 95% confidence intervals (CI), and partial *R* ^2^ for fixed effects with variances for random effects of individual and year, from global models of δ^13^C and δ^15^N values from brown bear *Ursus arctos* hair samples.

Fixed effect	δ^13^C		δ^15^N	
	*neighborhood* + *relative age* + *avg minimum age* + *day‐of‐year* + *sex* + (*relative age* × *sex*) + (1 | *individual*) + (1 | *year*)			
	Estimate (CI)	Partial *R* ^2^	Estimate (CI)	Partial *R* ^2^
(Intercept)	−15.27 (−19.06, −11.46)*	—	19.02 (13.87, 24.27)*	—
*neighborhood*	0.99 (0.52, 1.48)*	0.158	1.49 (0.82, 2.16)*	0.181
*relative age*	0.19 (0.08, 0.30)*	0.094	0.21 (0.07, 0.36)*	0.066
*avg minimum age*	−0.10 (−0.34, 0.14)	0.010	−0.09 (−0.43, 0.25)	0.004
*day‐of‐year*	−0.02 (−0.04, −0.01)*	0.055	−0.04 (−0.06, −0.01)*	0.076
*sex*	0.00 (−0.72, 0.71)	0.000	0.11 (−0.92, 1.14)	0.001
*relative age* × *sex*	0.07 (−0.14, 0.28)	0.003	0.20 (−0.08, 0.47)	0.004

Random effect	Variance (δ^13^C)	Variance (δ^15^N)
*individual*	0.256	0.560
*year*	0.078	0.170
Residual	0.791	1.381

*Note:* An asterisk indicates that the 95% confidence interval does not include zero, suggesting a statistically supported effect.

## 4 Discussion

Using stable isotope data from brown bear hair samples, we tested three hypotheses and detected another important source of variation in isotopic signals. First, as predicted, there was greater inferred consumption of salmon on one side of the lake than the other, consistent with the relative densities of salmon. Second, contrary to our prediction, there was no difference in isotopic signal between males and females. Third, as predicted, there was an increasing trophic position with the bear's age since first detection. Finally, we detected a decreasing inferred consumption of salmon over the ca. 6‐week sampling period within years.

The patterns detected were evident against a background of great variation among individuals in isotopic values, consistent with research on brown bears elsewhere in North America (e.g., Mowat and Heard 2006; Mangipane et al. 2020) and Japan (e.g., Jimbo et al. 2022). The overall averages we detected (δ^15^N: ~11 to 12, δ^13^C: ~−20) were comparable to those reported by Van Daele et al. (2013) for brown bears on Kodiak Island, where multiple species and runs of salmon are available to bears. The higher contribution of salmon to bear diets on the north side of the lake was consistent with the higher abundance, density, and predation on salmon in those three streams (Happy, Hansen, and Eagle creeks) compared to those on the south side (Bear, Yako, and Whitefish creeks). The number of salmon in each neighborhood (Table 1) and areas used by salmon in these streams (Quinn et al. 2003) produce estimates of 8735 salmon/ha in the north neighborhood and 2705 in the south, for a 3.23‐fold difference. Nevertheless, the shallow, narrow streams facilitate predation by bears on salmon (Quinn et al. 2003, 2017), and similar proportions of the salmon present were killed by bears (averaging 34.5% in the north area and 33.8% in the south). Estimates of the numbers of different bears vary among years (8–39 on the north side and 9–23 on the south side) but no significant difference in average abundance in 9 years from 2012 to 2022 (20.8 on the north side and 17.8 on the south side, t = 1.35, *p* > 0.2, two‐tailed test; data from Wirsing et al. (2018) and additional unpublished data). Thus, the higher inferred contribution of salmon to the diets on the north side was consistent with higher abundance of salmon per bear. Perhaps the lower absolute abundances and densities of salmon on the south side reduce the reliance of bears on them, though we have no information on the relative abundance of alternative dietary items.

The current, expanded data from our sites did not support our earlier finding that males had higher trophic positions than females (Ro et al. 2021), and thus contrast with findings that adult male brown bears consumed more meat, including salmon, than did females (Jacoby et al. 1999; Hobson et al. 2000; Van Daele et al. 2013; Mangipane et al. 2020). Mowat and Heard (2006) reported that higher meat consumption by males was most prevalent where meat was a smaller contribution to the diets, but where meat was a larger fraction of the diets, consumption tended to be more similar between sexes. Perhaps at our study streams, salmon are sufficiently abundant and accessible that factors besides sex (e.g., age, social status, wariness to humans) are more important in determining the diets of bears.

It is important to note two considerations when comparing the present results to those of other studies inferring diet or reliance on salmon from stable isotope samples. First, our samples were collected exclusively along the stream corridors, thus excluding any bears nearby that moved exclusively along upland trails. In contrast, other studies have included samples from bears killed by humans (e.g., Van Daele et al. 2013), passive hair snares (e.g., Adams et al. 2017), and those sampled as part of directed research programs (e.g., Belant et al. 2006). These and many other studies included samples from much larger areas and distances from spawning salmon, and much broader periods of the year compared to our work. These spatial and temporal factors might affect the comparability of our results to those of other studies.

In addition to the differences in spatial and temporal scope of our sampling and other studies, fewer males were included than females, despite our efforts to include males in the samples we processed. Over the decade of our entire study, we have consistently detected more females than males along these study streams. Genetic identification of 119 samples from 2012 to 2015 indicated 57% females and 43% males (Wirsing et al. 2018). Updated analysis through 2022 supported this pattern; more females were detected than males, and individual females were detected more often than individual males within a season (Wirsing, Waits, Adams and Quinn, unpublished data). Finally, Wold et al. (2020) found that females more often avoided the wires (collecting hair) than did males based on video analysis, so the genetic analysis likely underestimates the female skew in the data. Thus, males and females differ in abundance and behavior in this system, as elsewhere (e.g., Schooler et al. 2025), so the lack of difference in salmon consumption here does not necessarily contradict differences found in other areas.

The increasing trophic position and inferred consumption of salmon as individual bears age is consistent with evidence that adult brown bears consumed more meat, including salmon, compared to sub‐adults on Kodiak Island, Alaska (Van Daele et al. 2013), and in Japan (Jimbo et al. 2022). We do not know the bears' ages when they were first genetically detected and videos revealed that cubs routinely contacted the wires (Wold et al. 2020), so the samples likely represented a wide range of initial ages. Brown bears reach their asymptotic maximum body‐frame size at about 8–14 years of age but continue to grow in mass (Hilderbrand et al. 2018). Only nine of the individuals we sampled had minimum ages of 8 or more, so many of the bears were likely still in the period of rapid growth. Thus, the trophic enrichment we observed as individual bears aged is consistent with the idea that increasing size, and perhaps also experience, confers greater access to salmon. The difference was not great, however, perhaps because even young bears can capture and scavenge salmon in these streams (Lincoln et al. 2021; Bozzi et al. 2025). In contrast, the diets of bears sampled in other areas, where they depend more on terrestrial meat or have less access to salmon, might vary more with age.

We observed wide variation in nitrogen and carbon isotope signatures among bears, even though all samples were collected from wires directly across shallow streams with abundant salmon that bears kill and consume. Similarly, Edwards et al. (2011) reported great variation in diet within a population of brown bears in the Canadian Arctic, with δ^15^N values ranging from 2.0‰ to 11.0‰, which the authors characterized as “near‐complete herbivory to near‐complete carnivory” [p. 877]. Our samples indicated raw average δ^15^N values (i.e., not modeled to account for sources of variation) for individuals from 3.9‰ to 14.9‰ for females and 8.5‰–14.6‰ for males. We have not conducted formal vegetation surveys of the areas around the study streams, but berries are abundant and we routinely see bear scat with berries and other plant material, so foods representing lower trophic levels are available and commonly consumed. Most of the isotopic variation we detected among individuals likely arose from differences in behavior because there was no detectable effect of sex or average minimum age. For example, some bears may have been using the streams primarily for traveling corridors rather than foraging areas.

We detected a source of variation in our sampling unrelated to our hypotheses: the decrease in isotopic signature with date within the season when the samples were obtained. In these streams, the sockeye salmon timing is very regular and varies by only a few days among years in each stream, and the sequential arrival in the six streams is also very predictable, starting in Happy Creek in mid‐July, followed about a week later by Hansen, Yako and Bear creeks, and about another week later than spawn in Eagle and Whitefish creeks, and the bears follow this sequence (Quinn et al. 2014). Such sequential use of salmon is seen elsewhere, for example, on Kodiak Island (Barnes 1990; Deacy et al. 2016). The hair samples we obtained likely included some from the present season and some grown the previous year, given that molting is thought to occur between May and October (Jacoby et al. 1999; Schwartz et al. 2003; Jimbo et al. 2020; Stern et al. 2025). Hair from the previous year may be collected in higher proportions early in the season, as it is shed and would reflect the salmon consumed the previous year. In contrast, hair grown in the current year would primarily represent the diet prior to foraging on salmon in the stream. This might explain the general shift in isotopic signatures we observed over the season (i.e., mid‐July to late August). Thus, paradoxically, at the end of the salmon run, when bears have been feeding on salmon, the hair sampled from that year might primarily reflect their diet before salmon arrived.

The collection of multiple samples from known individuals within and among years, combined with data on salmon abundance, was an important asset of the study. Stable isotope studies of brown bears that have been killed (Van Daele et al. 2013; Matsubayashi et al. 2014) may reveal changes in the population's diet over extended periods (Hilderbrand et al. 1996; Matsubayashi et al. 2015) but typically have not revealed how consumption changes over the lives of individuals. We note, however, the recent development using eye lens samples to reconstruct diet shifts over a bear's ontogeny (Miura et al. 2025). Telemetry studies can allow repeat sampling, but often for only one (Mangipane et al. 2018) or a few years (Belant et al. 2006), and other studies are conducted over such large areas that it is difficult to know how many salmon were available to specific bears (Adams et al. 2017; Rogers 2021).

The overall patterns in the data were clear, but there are some methodological considerations, including the turnover of hair mentioned above. Our samples were obtained passively by bears that encountered wires across the streams and left hair on the barbs. Videos indicated that bears contacted the wires in > 80% of the times they approached them, though females more often avoided wires than males did (Wold et al. 2020), and bears did not become less likely to encounter wires as years passed (Lincoln et al. 2020). Thus, while the sampling was imperfect, it generally represented the bears using the streams. As noted in the Methods, the subsample of hairs that we processed for stable isotopes was not randomly selected from those processed for DNA. We tried to balance sexes, sides of the lake, multiple study years, and individuals detected over many years.

The great variation in salmon use among bears with ready access to them documented here adds to the growing literature on the importance of individuals in behavioral ecology (Bolnick et al. 2003). Our evidence that individual bears sampled on salmon‐bearing streams ranged from almost complete reliance on plants to near‐complete reliance on meat suggests great differences in their ecological roles. By implication, bear populations with access to salmon may not all respond the same way to changes in the abundance of salmon or other key resources.

## Author Contributions

**Thomas P. Quinn:** conceptualization (equal), investigation (equal), project administration (equal), supervision (equal), writing – original draft (equal), writing – review and editing (equal). **Jennifer H. Stern:** conceptualization (equal), data curation (equal), formal analysis (equal), writing – original draft (equal), writing – review and editing (equal). **N. Grace Henry:** data curation (equal), formal analysis (equal), investigation (equal), methodology (equal), writing – review and editing (equal). **Annika K. McFeely:** investigation (equal), methodology (equal), writing – review and editing (equal). **HyeJoo Ro:** data curation (equal), investigation (equal), methodology (equal), writing – review and editing (equal). **Blaise Stricker:** investigation (equal), methodology (equal), writing – review and editing (equal). **Liz Voytas:** investigation (equal), methodology (equal), writing – review and editing (equal). **Lisette P. Waits:** methodology (equal), project administration (equal), writing – review and editing (equal). **Jennifer R. Adams:** data curation (equal), investigation (equal), methodology (equal). **Aaron J. Wirsing:** conceptualization (equal), data curation (equal), investigation (equal), methodology (equal), project administration (equal), supervision (equal), writing – original draft (equal), writing – review and editing (equal).

## Conflicts of Interest

The authors declare no conflicts of interest.

## Supporting information


**Table S1:** Provenance of all brown bear hair samples analyzed as part of this isotopic study. Each hair sample is associated with an individual bear id, the stream and stream trio (i.e., neighborhood) where it was collected (Happy, Hansen, Eagle, HHE; Bear, Yako, Whitefish, BYW), the date, day‐of‐year, and year of collection, the sex of the bear from which it was collected, δ15N and δ13C values, and the minimum age of the bear from which it was collected (i.e., the year when a given sample was obtained for stable isotope analysis, relative to the year when the first sample from the same bear was obtained).

## Data Availability

The stable isotope data for individual bears, with collection site and date, are included in the Supporting Information for this paper as Table S1. The full dataset, metadata, and R code have been publicly archived on figshare (https://doi.org/10.6084/m9.figshare.31302703.v1).
